# Comment on "Oncovascular surgery"

**DOI:** 10.1590/1806-9282.20241479

**Published:** 2025-03-31

**Authors:** Veronica Maggi, Simone Garzon, Massimo Piergiuseppe Franchi, Gian Franco Veraldi, Stefano Uccella

**Affiliations:** 1 University of Verona, Azienda Ospedaliera Universitaria Integrata Verona, Department of Surgery, Dentistry, Pediatrics, and Gynecology, Unit of Obstetrics and Gynecology – Verona, Italy.; 2 University of Verona, AUOI Verona, Unit of Vascular Surgery – Verona, Italy.

Dear Editor,

We read the article "Oncovascular Surgery" by Gomes et al. with great interest. They focused on the complexity of resecting major vessels in surgeries for vessel-origin tumors, intravenous leiomyomatosis, and retroperitoneal soft tissue sarcoma. They highlighted the fundamental role of vascular surgeons in planning vascular resections and treating vascular complications of these surgeries^1^. Although there is still scarce literature about the long-term oncological benefits of such operations, a growing body of evidence has shown that vascular surgery with major vessel resection in advanced pancreatic cancer, renal cancer, and retroperitoneal sarcomas is associated with improved survival^2-6^. In this scenario, we would like to add our experience in the resection of major vessels during surgery for gynecological cancers and the impact of these procedures. We recently published the largest available case series of planned ultraradical vascular resections performed to obtain complete cytoreduction during surgery for advanced or relapsing ovarian cancer, with an updated review of the literature on this topic^7^. We reviewed data of all ovarian cancer patients who underwent primary, interval, or secondary debulking surgery at our Institution between January 2021 and January 2023. All patients who underwent resection, with or without reconstruction, of one or more major vessels during ovarian cancer surgery were identified; non-ovarian primary malignancies were excluded. We defined as major vessels the vena cava, the aorta, the common iliac vessels (either vein or artery), and the external iliac vessels (either vein or artery). In every case, the definitive pathology confirmed the diagnosis of ovarian cancer and the infiltration of the vessel. In the systematic review of the literature, we identified only seven previously described ovarian cancer patients who underwent either primary cytoreduction, interval debulking surgery, or secondary cytoreduction with concomitant major vessel resection (aorta, inferior vena cava, common or external iliac vessels) with or without reconstruction. Our study showed, consistent with the study by Gomes et al., that the involvement of a multidisciplinary team of experts in complex radical procedures allows the performance of planned major vascular resection with an acceptable risk of complications during debulking surgery for gynecological malignancies. The multidisciplinary approach allows the complete eradication of the disease from areas previously deemed inoperable. Reaching resection rates of no residual tumor in gynecological oncological patients with advanced disease is paramount to increasing disease-free survival, overall survival, and quality of life. Therefore, referring patients to centers with comprehensive experience in the pre-, intra-, and post-operative management of these complex radical procedures is crucial to guarantee the best treatment^8^.
